# The discriminant validity of single-question assessments of subjective cognitive complaints in an Asian older adult population

**DOI:** 10.3389/fnagi.2022.901592

**Published:** 2022-08-08

**Authors:** Ting Pang, Xuhao Zhao, Xindi He, Cheuk Ni Kan, Narayanaswamy Venketasubramanian, Ching-Yu Cheng, Changzheng Yuan, Christopher Chen, Xin Xu

**Affiliations:** ^1^School of Public Health & the 2nd Affiliated Hospital of School of Medicine, Zhejiang University, Hangzhou, China; ^2^Memory, Ageing and Cognition Centre, Department of Pharmacology, Yong Loo Lin School of Medicine, National University of Singapore, Singapore, Singapore; ^3^Singapore Eye Research Institute, Singapore, Singapore

**Keywords:** single-question assessment, subjective cognitive complaints, dementia, cognitive screening, discriminant validity

## Abstract

**Objective:**

To compare the discriminant validity of three different single-question assessments of subjective cognitive complaints (SCC) for dementia in a community-based older adult population in Singapore.

**Methods:**

Eligible older adults aged ≥60 were recruited into phase I for identifying those who require further assessment using the Abbreviated Mental Test (AMT) and progressive forgetfulness question (PFQ). Participants who failed either tests entered phase II and were administered various single-question assessments of SCC, such as the 8th question on the patient Ascertain Dementia 8 (AD8-8_pt_), informant AD8 (AD8-8_info_), and the 10th item on the Geriatric Depression Scale (GDS-10), followed by the Montreal Cognitive Assessment (MoCA) and a formal neuropsychological battery to identify the participant’s cognitive status by a research diagnosis and DSM-IV criteria. Differences in characteristics among diagnostic groups were compared. All discriminatory indices (sensitivity, specificity, positive, and negative predictive values, overall accuracy) for these single-question assessments and their combinations with the MoCA were calculated and reported to confirm their discriminant validity in identifying the existence of subjective complaints and objective impairment.

**Results:**

A total of 3,780 participants were assessed at phase I, of which 957 entered and completed phase II. Of whom, 911 were dementia-free and 46 had dementia. The MoCA (13/14) displayed good sensitivity (95.6%), specificity (81.5%), and overall accuracy (82.1%) for dementia detection. The GDS-10 and AD8-8_pt_ showed poor discriminant validity, while the AD8-8_info_ had the highest specificity (83.2%) and the greatest overall accuracy (82.5%) for dementia. Compensatory combination of the AD8-8_info_ with MoCA, the sensitivity and positive predictive values were optimized (100%), while the conjunctive combination of two tools achieved excellent specificity (96.3%) and overall accuracy (94.8%) in discriminating dementia patients.

**Conclusion and implications:**

Combining a reliable single-question SCC assessment with an objective tool can efficiently discriminate dementia patients from healthy older adults in the community.

## Introduction

Dementia is one of the top causes of death among all diseases. Currently, more than 55 million people live with dementia worldwide and there are nearly 10 million new cases every year (World Health Organization, 2021). The increase in dementia cases has caused a serious challenge for the health system and society. Although it mainly affects older people, it is not an inevitable consequence of aging. Studies have shown that early detection of cognitive impairment prior to the occurrence of dementia could benefit early management and intervention for at-risk older adults, to delay the process of cognitive decline and prevent dementia onset (Livingston et al., 2020). Hence, early screening for dementia in the community is particularly essential.

Subjective cognitive complaints (SCC), also known as subjective cognitive decline, or subjective memory complaints (Rabin et al., 2017), a key sign of preclinical Alzheimer’s Disease dementia, refers to a persistent decline in memory and/or other cognitive abilities reported by individuals or informants in the absence of objective neuropsychological evidence (Jessen, 2014). SCC is common among the older adults, and its prevalence increases with advancing age. Subjects with SCC are at a high-risk conversion to mild cognitive impairment and dementia, especially those aged over 75 (Jessen et al., 2020; Slachevsky et al., 2020). While objective cognitive assessments remain the gold standard for assessing cognitive function, which assesses the cognitive performance at a single point in time, such as the Mini-mental State Examination (MMSE), the Montreal Cognitive Assessment (MoCA), and comprehensive cognitive batteries, SCC assessments can be used to determine the existence of subjective complaints from participants and capture longitudinal cognitive changes (Jessen, 2014). Hence, using a self-reported SCC assessment as an additional tool in a large-scale cognitive screening may be an easy and more cost-beneficial way to identify those at-risk for cognitive decline and dementia (Wasef et al., 2021).

Among all SCC assessments, the use of a single-question format of SCC assessment has been introduced and popularized. There is growing evidence to confirm that SCC, as assessed by single-question tools as well as more comprehensive tools, was associated with an increased risk of cognitive decline and dementia (Jungwirth et al., 2008; Rönnlund et al., 2015; Rabin et al., 2017; Peters et al., 2019). The progressive forgetfulness question (PFQ) was reported a simple but effective in screening for dementia in a primary care setting in Singapore, by ruling out people at lower risk of dementia (Chong et al., 2006). Similarly, the Hypertension in the Very Elderly (HYVET) Trial examined the role of the 10th item on the geriatric depression scale (GDS) “do you feel have more problems with memory than most?” in predicting incident dementia in a hypertensive older population and found that baseline SCC was associated with an increased risk of developing any dementia (Peters et al., 2019). The England and Wales Departments of Health Commissioning for Quality and Innovation (CQUIN) strategy initiative have recommended and implemented the use of a single-question as the first step in the assessment pathway in large-scale dementia screening (Kmietowicz, 2012; Hendry et al., 2014).

Although these studies have highlighted the applicability of such SCC assessments for large-scale use, they did not compare such SCC assessments with gold standard neurocognitive evaluation, hence could not ascertain the discriminant validity of such brief tools for dementia screening purposes. Thus, there is a need for further validation of the single-question assessments of SCC in a large population of community-dwelling older adults.

Hence, the present study aimed to (1) explore the discriminant validity of single-question assessments of SCC for dementia detection in an Asian older adult population; (2) examine whether the combination of single-question SCC assessments with a structured cognitive tool (MoCA) could improve the discriminant indices for dementia detection. We hypothesized that a single-question SCC performed by the participants, or their caregivers can quickly identify those at higher risk of developing dementia and who would benefit more from a detailed cognitive assessment. Secondly, the single-question SCC can improve discriminant indices when used with in combination with the MoCA.

## Materials and methods

### Study design

The Singapore Epidemiology of Eye Diseases study (SEED) was conducted in multi-ethnic subjects aged 60 years or older living in the community in Singapore. Community residents of three ethnic groups (Chinese, Malays, and Indians) were recruited from the baseline participant pool by telephone or home visits between 2011 and 2017. The details of the SEED study have been previously reported (Majithia et al., 2021). The SEED study had two phases, with the phase I consisting of a questionnaire administered by trained investigators on the participants’ demographic information and relevant risk factors, along with a primary screening of participants’ cognitive function using the Abbreviated Mental Test (AMT) and PFQ (Sahadevan et al., 1997). The optimal cut-off of AMT adjusted for education is 6/8 (screen positives were defined as AMT score ≤6 among those with ≤6 years of formal education, or AMT score ≤8 among those with >6 years of formal education), which has been previously validated in Singapore (Sahadevan et al., 2000). The PFQ is a single format question by asking participants or their informants (“Do you/he/she have progressive forgetfulness”), and those who answer YES is considered positive (Chong et al., 2006). Participants who failed on either the AMT or/and PFQ tests were tested positive and hence invited to the second phase of the study. In the phase II, participants underwent a set of single-question SCC assessments, such as GDS-10 and AD8-8, followed by the MoCA and a comprehensive neuropsychological evaluation (Xu et al., 2016a). Details on the SEED study procedures can be found elsewhere (Hilal et al., 2013, 2017; Wong et al., 2019).

### Study participants

At phase I, a total of 3,780 individuals completing both the AMT and/or the PFQ assessments, of whom 1,593 were screened positive and 957 underwent comprehensive cognitive and clinical investigations in phase II.

### Single-question subjective cognitive complaints assessments

Three single-questions for assessing SCC were used:

**Table:** 

Single-question SCC	Respondent	Question
GDS-10^a^	Participants	Do you feel have more problems with memory than most?
AD8-8_pt_^b^	Participants	Do you have daily problems with thinking and/or memory?
AD8-8_info_^c^	Main caregivers	Does the participant/patient have daily problems with thinking and/or memory?

^*a*^GDS-10, the 10th item of Geriatric Depression Scale.

^*b*^AD8-8_pt_, the 8th item of patient AD8.

^*c*^AD8-8_info_, the 8th item of informant AD8.

### Cognitive assessments and dementia diagnosis

Brief and comprehensive cognitive assessments were administered to all participants in phase II. The MoCA was performed, followed by a formal neuropsychological battery (the vascular dementia battery, VDB) (Narasimhalu et al., 2011; Xu et al., 2016b), which was locally validated for Singaporean older adults (Hilal et al., 2013). Dementia was diagnosed according to the DSM-IV criteria (American Psychiatric Association, 1994), by consensus at formal meetings of the research team.

### Statistical analyses

Demographic characteristics and cognitive outcomes were presented as mean ± SD, number with/without number of cases (%) as appropriate. One-way ANOVA and chi-square tests were used to compare differences of sample characteristics by the cognitive outcome. Furthermore, Bonferroni correction was applied for multiple comparisons between groups, and that a *p*-value of <0.016 was considered statistically significant.

In addition to sensitivity and specificity, discriminant validity of the tools was estimated by positive predictive value (PPV) and negative predictive value (NPV). PPV and NPV are defined as a proportion of people with a positive/negative result who actually have/do not have the disease. A higher PPV in the observed population signifies less false positives and a higher NPV will have small number of false negatives (Chu, 1999; Stojanovic et al., 2014).


Sensitivity=True⁢Positive/(True⁢Positive+False⁢Negative)

Specificity=True⁢Negative/(True⁢Negative+False⁢Positive)

PPV=True⁢Positive/(True⁢Positive+False⁢Positive)

NPV=True⁢Negative/(True⁢Negative+False⁢Negative)

Accuracy=(TruePositive+TrueNegative)/(TruePositive

+False⁢Positive+True⁢Negative

+FalseNegative)

The discriminant indices of dementia were calculated for GDS-10, AD8-8_pt_, AD8-8_info_, and MoCA (using the optimal cut-off points) were calculated using the above formula separately. The Pearson correlation coefficient and Cohen’s kappa coefficient were calculated for different single-question SCC tools. Compensatory and conjunctive combinations of the single-question SCC with MoCA were employed to determine if combination approaches would enhance the discriminatory values over MoCA alone. Compensatory combination requires either test to be positive, whereas conjunctive combination requires both tests to be positive. Compensatory combination generally improves sensitivity whereas conjunctive combination generally improves specificity (Kan et al., 2019).

All analyses were done on IBM SPSS.26.0, and a *p*-value < 0.001 was considered statistically significant.

## Results

### Demographic data

**FIGURE 1:**
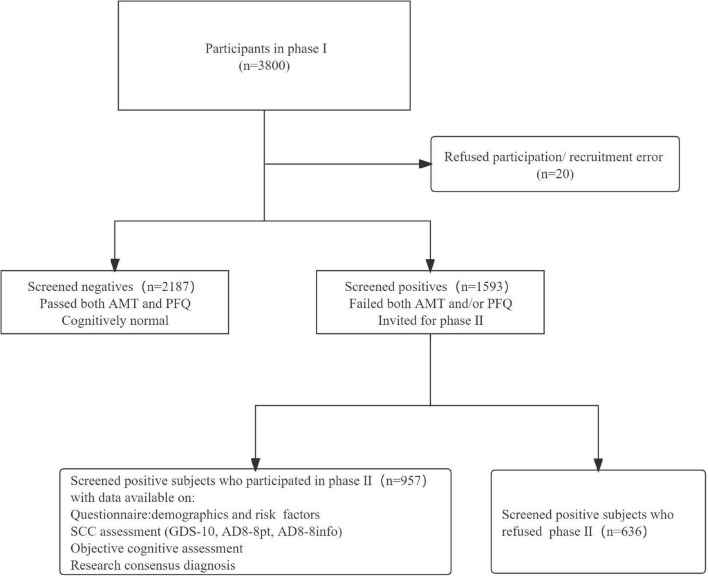
Study recruitment.

Figure 1 shows the study recruitment flow chart. A total of 957 participants were included in the final analysis, 46 (4.8%) were diagnosed with dementia. Compared with those who were diagnosed as dementia-free subjects, those who were diagnosed as dementia were older (mean age 78.8 year vs. 69.8 year), more often female (78.3% vs. 50.4%), have lower education levels (91.3% vs. 61.0%), and that these differences were statistically significant. We also find a notable difference in ethnicity between the two groups. Sample characteristics at phase II are shown in Table 1.

**TABLE 1:** Sample characteristics at Phase II.

Characteristics	Dementia (*n* = 46)	Dementia-free (*n* = 911)	Total (*n* = 957)	*P*-value
Age (mean, SD)	78.8 ± 5.6	69.8 ± 6.4	70.2 ± 6.6	<0.001
Gender, female (*n*%)	36 (78.3)	459 (50.4)	495 (51.7)	<0.001
Education, 0–6 years (*n*%)	42 (91.3)	556 (61.0)	598 (62.5)	–
Ethnicity	–	–	–	0.001*
Chinese (*n*%)	7 (15.2)	293 (32.2)	300 (31.3)	–
Malay (*n*%)	27 (58.7)	296 (32.5)	323 (33.8)	–
Indian (*n*%)	12 (26.1)	322 (35.3)	334 (34.9)	–
GDS-10^a^ (yes, *n*%)	18 (40.9)	155 (17.0)	173 (18.6)	<0.001
AD8-8_pt_^b^ (yes, *n*%)	15 (39.5)	194 (23.4)	209 (24.1)	<0.001
AD8-8_info_^c^ (yes, *n*%)	27 (67.5)	123 (16.7)	150 (19.3)	<0.001
MoCA^d^ (mean, SD)	8.3 ± 4.1	19.4 ± 5.1	18.8 ± 5.6	<0.001

^*a*^GDS-10, the 10th item of Geriatric Depression Scale.

^*b*^AD8-8_pt_, the 8th item of patient AD8.

^*c*^AD8-8_info_, the 8th item of informant AD8.

^*d*^MoCA, the Montreal Cognitive Assessment. *The differences are significant after Bonferroni correction.

### Discriminant validity of single-question subjective cognitive complaints

Table 2 summarizes all discriminant indices of the different single-question SCC tools and MoCA for detecting dementia. Results showed that the GDS-10 and AD8-8_pt_ had low sensitivity and moderate specificity, while the AD8-8_info_ had the highest specificity (83.2%) and the greatest overall accuracy (82.5%), although all SCC questions showed a high NPV (>95%) and low PPV (<20%). At an optimal cut-off of 13/14, MoCA displayed good sensitivity (95.6%) and specificity (81.5%). All three single-question SCC tools have poor agreement among each other (Supplementary Tables 1, 2). There was a statistically significant difference in the proportion of endorsement on the three SCC questions, whereas the AD8_pt_ has more endorsement than the other tools (Supplementary Figure 1).

**TABLE 2:** Discriminant indices of the different tools for detecting dementia.

Brief tools	Sensitivity (%)	Specificity (%)	PPV (%)	NPV (%)	No of cases correctly identified	No of healthy subjects correctly identified	Overall Accuracy (%)
GDS-10^a^	40.9	82.9	10.4	96.7	18/44	756/911	80.0
AD8-8_pt_^b^	41.7	76.5	7.2	96.8	15/36	631/825	75.0
AD8-8_info_^c^	69.2	83.2	18.0	98.1	27/39	609/732	82.5
MoCA^d^ (cut-off: 13/14)	95.6	81.5	20.3	99.7	43/45	742/911	82.1

^*a*^GDS-10, the 10th item of Geriatric Depression Scale.

^*b*^AD8-8_pt_, the 8th item of patient AD8.

^*c*^AD8-8_info_, the 8th item of informant AD8.

^*d*^MoCA, the Montreal Cognitive Assessment.

### Combined utility of the Montreal cognitive assessment and single-question subjective cognitive complaints tools for detecting dementia

Subsequently, we explored whether the combination of the MoCA with another single-question SCC tool can improve the discriminant indices. The compensatory combination of MoCA and AD8-8_info_ reached an optimal sensitivity and PPV. The specificity of MoCA can be increased to 96.3% and 96.2% by combining with the AD8-8_info_ and GDS-10 in a conjunctive manner, respectively. Also, the overall accuracy of this conjunctive combination was improved to 94.8% and 93.6% (Table 3).

**TABLE 3:** Discriminant indices of the combination of single-question subjective cognitive complaints (SCC) with Montreal cognitive assessment (MoCA) for detecting dementia.

Combination of brief tools	Sensitivity (%)	Specificity (%)	PPV (%)	NPV (%)	No of cases correctly identified	No of healthy subjects correctly identified	Overall accuracy (%)
MoCA^a^ or GDS-10^b^	95.6	68.3	13.0	99.7	43/45	622/911	69.6
MoCA and GDS-10	40.9	96.2	34.0	97.1	18/44	876/911	93.6
MoCA or AD8-8_pt_^c^	97.8	63.4	12.4	99.8	44/45	536/846	65.1
MoCA and AD8-8_pt_	36.8	94.1	20.9	97.2	14/38	840/893	91.7
MoCA or AD8-8_info_^d^	100	66.2	14.8	100	45/45	508/767	68.1
MoCA and AD8-8_info_	62.5	96.3	43.1	98.3	25/40	849/882	94.8

^*a*^MoCA, the Montreal Cognitive Assessment.

^*b*^GDS-10, the 10th item of Geriatric Depression Scale.

^*c*^AD8-8_pt_, the 8th item of patient AD8.

^*d*^AD8-8_info_, the 8th item of informant AD8.

## Discussion

In this study, we found that the discriminant indices of all single-question SCC assessments, such as the GDS-10, AD8-8_pt_, and AD8-8_info_, were inferior to the MoCA for dementia detection. However, combining an SCC assessment (AD8-8_info_) with an objective tool (MoCA) maximized the discriminant validity of dementia detection.

Our study showed that, although feasible, using a single-question of SCC itself does not yield optimal discriminant validity for dementia screening, which is consistent with other studies (Eichler et al., 2015). The finding from our present study also reaffirms the importance of objective cognitive assessments in dementia screening, as most older people are unable to make an accurate assessment of their cognitive performance, even though SCC has some predictive value. Moreover, some participants with depressive symptoms may exaggerate their subjective memory complaints but not have objective cognitive decline, which can lead to false positive results (Brailean et al., 2019). Therefore, using such a single-question SCC assessment alone may be difficult to achieve case detection in large-scale screening, especially in community populations.

We found that using a combination of objective tools with a single-question SCC can maximize sensitivity (compensatory) and specificity (conjunctive). The optimal cut-off of the full version of MoCA (13/14) for dementia detection in our study is indeed lower than other reports, but was consistent with the previous studies in Singapore, possibly due to the generally low level of education in the Asian population (62.5% with 0–6 years of education) (Chan et al., 2015; Phua et al., 2018; Kan et al., 2019). When combined in a compensatory manner, the AD8-8_info_ with MoCA, the sensitivity, and PPV is optimized (100% in our study), which allows the inclusion of as many patients as possible in a large-scale dementia screening. In addition, the conjunctive approach of the AD8-8_info_ with MoCA showed improvement in specificity (96.3%) and overall accuracy (94.8%), which helps to narrow the screening pool and exclude as many healthy people as possible, while also saving time and human resources. From the findings of the present study, the use of a single-question SCC tool should be used in combination with an objective cognitive assessment test in large-scale dementia screening in the community.

Evidence showed that a combination approach can improve the utility of cognitive tests in dementia screening. According to that the priority of the two combination strategies, a compensatory combination is capable to enhance the overall sensitivity, while a conjunctive combination may improve PPV (Chan et al., 2016). Combination strategies were usually based on specific research settings and objectives. In clinical settings, where patients were referred from somewhere else due to memory complaints may benefit from a compensatory combination to optimize screening sensitivity. However, in the community setting, the conjunctive combination approach may be preferred to achieve better PPV and reduce false positives. This approach will also help facilitate and reserve resources in community healthcare systems, where screening infrastructure and resource is scarce (Iliffe et al., 2009). Meanwhile, before the structured objective cognitive assessment, adding a single-question SCC assessment can establish a good relationship with the participants and relieve their tension.

We found that the discriminant indices of AD8-8_info_ were superior to other SCC assessments, including AD8-8_pt_. Moreover, we can see that using the 8th item of AD8, 67.5% of informants reported memory problems with their study partners among dementia participants, while only 39.5% of patients self-reported subjective memory problems. Similarly, in the dementia-free group, the proportion of informants who correctly reported no memory decline was slightly higher than that of participants (83.3% vs. 76.6%). This result is consistent with the previous studies which showed asking informants are more reliable than subjects, particularly noticeable among patients with dementia (Yim et al., 2017; Kan et al., 2019). It could be that dementia is a progressive neurodegenerative disease; many old people do not have an accurate assessment of their cognitive abilities, especially those who have already shown symptoms of cognitive decline. In contrast, informants can capture such progressive changes because of regular interaction with the subject. Meanwhile, the informant confirmation is a key feature of clinical cognitive decline and might be a better predictor of objective performance as disease severity progresses (Morrison et al., 2022). Although such informant-based tools may be affected by individual differences of caregivers, such as familiarity between caregivers and subjects, reliability of answers, etc. These problems can be well solved by combining them with objective cognitive tools.

It should also be mentioned that the cognitive changes observed by various SCC tools are different. In terms of the implications of the SCC questions, the GDS-10 asks about one’s memory problems compared to most people, which is a “cross-sectional” comparison; while the AD8-8 asks about changes in memory over recent years, which is a longitudinal comparison. Hence, during implementation phase, the SCC assessments need to be carefully selected according to the type of interviewees (Diaz-Galvan et al., 2021; Morrison et al., 2022).

The strength of this study is that it is a community-based research design and has a large sample size covering different ethnic groups in Singapore. The second strength was the inclusion of multiple single-question SCC assessments in the present study which enabled the comparison among varying tools. The third strength was the use of a comprehensive objective cognitive assessment which provided the diagnosis of a spectrum of cognitive function in the present study.

Several limitations require acknowledgment. First, the gold standard diagnosis of dementia was only administered in a subset of individuals who were screened positive in phase I, which may have result an underestimation of the prevalence of cognitive impairment. Also, due to the small number of dementia cases, we did not perform further studies on the subtypes of dementia. Future studies could target at preclinical stages of dementia, as well as different dementia subtypes. Secondly, our study was conducted in a community-based population in Singapore, the proportion of people who refused to participate in a comprehensive cognitive assessment was high (39.9%), hence more prone to selection bias. Besides, as the present study was conducted in the community, although the prevalence of dementia was consistent with other literature, the generally lower prevalence of dementia may have affected the estimation of PPV and NPV of cognitive screening tools (Chu, 1999). Future studies could further adjust the actual predictive values of these SCC tools according to the census data.

## Conclusion

This study demonstrated that using a combination of objective tools with the 8th question on the informant AD8 as a single-question SCC measure can maximize discriminant capacity for dementia detection in the community. Future studies are warranted to examine if single-question SCC measures can predict pathology-related cognitive changes among older adults at-risk of dementia.

## Data availability statement

The original contributions presented in this study are included in the article/Supplementary material, further inquiries can be directed to the corresponding author.

## Ethics statement

The studies involving human participants were reviewed and approved by the Singapore Epidemiology of Eye Diseases study (SEED) was approved by the National Healthcare Group Domain Specific Review Board. The patients/participants provided their written informed consent to participate in this study.

## Author contributions

XX and CC designed the study, developed the protocol, and obtained the ethics of this study. TP and XZ performed the data analysis and wrote the manuscript. XH, CK, NV, CC, and CY revised the manuscript. All authors approved of the final version of this manuscript.
