# Supplementary material for: The discriminant validity of single-question assessments of subjective cognitive complaints in an Asian older adult population
Source: Front Aging Neurosci. 2022 Aug 8;14:901592. doi: 10.3389/fnagi.2022.901592 (PMC9393535; doi:10.3389/fnagi.2022.901592)
Supplement: Supplementary file 1 [file Data_Sheet_1.docx]

**Supplementary materials**

**Table S1**

*Correlation Analysis of Single-question SCC Tools*

| Single-question SCC | GDS-10 | AD8-8info | AD8-8pt |
| --- | --- | --- | --- |
| AD8-8pt | 0.007 | -0.007 | 1.000 |
| AD8-8info | -0.044 | 1.000 | -0.007 |
| GDS-10 | 1.000 | -0.044 | 0.007 |

**Table S2**

*Consistency Analysis of Single-question SCC tools*

| Single-question SCC | GDS-10 | AD8-8info | AD8-8pt |
| --- | --- | --- | --- |
| AD8-8pt | 0.201^*^ | 0.351^*^ | / |
| AD8-8info | 0.119 | / | 0.351^*^ |
| GDS-10 | / | 0.119 | 0.201^*^ |

Note.^*^Differences significant at P＜0.01.

**Figure S1**

*Distribution of Different Responses to Single-question SCC Tools*

Note. P＜0.001 among these three SCC tools.


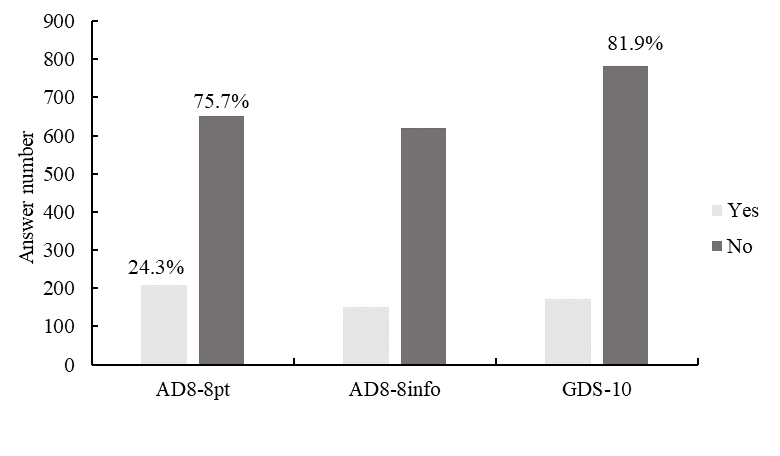


19.5%

80.5%

18.1%
